# Frequency and Type of Hepatic and Gastrointestinal Involvement in Juvenile Systemic Lupus Erythematosus

**DOI:** 10.1155/2017/8097273

**Published:** 2017-11-29

**Authors:** Leila Tahernia, Hosein Alimadadi, Fatemeh Tahghighi, Zahra Amini, Vahid Ziaee

**Affiliations:** ^1^Growth and Development Research Center, Tehran University of Medical Sciences, Tehran, Iran; ^2^Children's Medical Center, Pediatrics Center of Excellence, Tehran, Iran; ^3^Department of Pediatrics, Tehran University of Medical Sciences, Tehran, Iran; ^4^Pediatric Gastroenterology and Hepatology Research Center, Tehran University of Medical Sciences, Tehran, Iran; ^5^Pediatric Rheumatology Research Group, Rheumatology Research Center, Tehran University of Medical Sciences, Tehran, Iran

## Abstract

**Background:**

Systemic lupus erythematosus (SLE) is a frequent rheumatology disorder among children. Since hepatic involvement is a common systemic manifestation in lupus, the frequency and type of hepatic involvement were determined in pediatric cases of SLE admitted to Children's Medical Hospital from 2005 to 2014.

**Methods and Patients:**

In this observational case-series study, 138 pediatric cases of SLE were admitted in Children's Medical Center (a pediatric rheumatology referral center in Tehran, Iran) enrolled from 2005 to 2014 and the outcomes, frequency, and type of hepatic involvement were assessed among them.

**Results:**

Hepatic involvement was reported in 48.55% of total SLE patients. Aspartate aminotransferase (AST), alanine aminotransferase (ALT), and both enzymes higher than normal upper limits were detected in 8.7%, 5%, and 34.7% of lupus patients, respectively. Increased level of liver enzymes was categorized as less than 100, between 100 and 1000, and more than 1000 levels in 23.1%, 23.1%, and 2.1% of cases. The only gastrointestinal involvement in lupus patients contributing to hepatic involvement was gastrointestinal bleeding. Rising in liver enzymes was detected mostly in lupus patients without gastrointestinal bleeding (52.2% without versus 25.8% with gastrointestinal bleeding, *P* = 0.007).

**Conclusion:**

Approximately half of the pediatric patients suffering from SLE have hepatic involvement. No significant correlation was observed between various organs involvement and abnormal level of liver enzymes.

## 1. Introduction

Since systemic lupus erythematous (SLE) is an autoimmune multisystem disease, early diagnosis before the involvement of major organs is crucial. Involvement of kidney, central nervous system, and liver would result in numerous problems such as dialysis and cirrhosis [1, 2]. The liver involvement may be life-threatening [3]. Hepatic involvement in lupus could be the result of various factors including lupus hepatitis, medications during management of SLE (NSAIDs, Aspirin, immune-suppressors, hydroxyzine, hydroxychloroquine), fatty liver (due to corticosteroids), associated autoimmune hepatitis, primary biliary cirrhosis, cholangitis, and viral hepatitis [4].

In previous studies, the hepatic involvement and increase in liver enzymes in lupus patients had been reported mainly due to medications in the management of patients [5]. Since hepatic involvement could be primary or secondary in SLE, lupus hepatitis could be considered only after ruling out secondary causes and other etiological factors [6–9]. However, the majority of studies had assessed secondary causes of hepatic involvement in SLE [6, 9]. Primary hepatic involvement had rarely been studied in lupus [7, 8]. Moreover, most studies had been conducted mainly in the adult population [6, 7, 9]. Hepatic involvement in lupus is a challenging issue in rheumatology specially difference between lupoid (or autoimmune) hepatitis and overlap SLE with autoimmune liver diseases [10, 11].

Hepatic involvement in lupus is associated with higher mortality rate in children [12]. SLE disease is multisystem and rapidly progressive in childhood cases; so, a study on hepatic and gastrointestinal involvement in pediatric cases may be helpful in reduction of lupus burden especially with the absence of this involvements in diagnostic criteria. Accordingly, in this study frequency and type of hepatic involvement were evaluated among childhood-onset SLE admitted in a pediatric rheumatology referral center in Iran since 2005 to 2014.

## 2. Methods and Materials

In this observational study which was performed as a case-series, 138 consecutive pediatric cases of SLE admitted in Children's Medical Center, Pediatric Center of Excellence, Tehran, Iran, were enrolled by a census manner from 2005 to 2014. The outcomes, frequency, and type of hepatic involvement were assessed as the main purpose of this study, but other gastrointestinal (abdominal pain, gastrointestinal bleeding, pancreatitis, gastritis, esophagitis, cholestasis, etc.) and major organ involvements (cardiac, cerebral, renal, etc.) were evaluated as minor objectives. Lupus cases were diagnosed based on the presence of 4 of 11 American College of Rheumatology (ACR) criteria. ACR SLE classification criteria were revised and validated by Systemic Lupus International Collaborating Clinics (SLICC) group in 2012. According to SLICC, a person is classified as having SLE in presence of biopsy-proven lupus nephritis with ANA or anti-ds-DNA antibodies or 4 of diagnostic criteria, including at least one clinical and one immunologic criterion 2 [13].

Hepatic involvement due to underlying lupus disease was considered as lupus hepatopathy after ruling out all other medical problems that could result in the elevated level of aspartate aminotransferase (AST) and alanine aminotransferase (ALT), with normal level of alkaline phosphatase (ALP) and ultrasound assessment. Gastrointestinal endoscopic study was performed in all patients with gastrointestinal bleeding. Cholestasis was detected by the increased level of alkaline phosphatase (ALP) and gamma-glutamyltransferase (GGT). Liver ultrasound study was carried out in all patients in order to rule out local liver problems such as hepatic steatosis, granuloma, and cholestasis. Improvement in liver enzymes by medication withdrawal was considered as drug-induced hepatotoxicity.

All data were extracted from clinical records of lupus patients in hospital archives. Medical information was inserted in related forms.

Data analysis was performed on 138 childhood-onset SLE cases by SPSS (version 13.0). Chi-Square, Fisher, and Independent-Sample *T* tests were used for analyzing data. They were considered statistically significant at *P* values less than 0.05.

## 3. Results

Mean age at diagnosis was 8.45 ± 3.9 years. Patients were prominently female (79%). Jaundice, cholestasis, and hepatomegaly were considered in 3.6%, 0.7%, and 28.2%, respectively. Hepatic involvement was detected in 48.55% including one patient with increased hepatic enzymes induced by medications (prescribed for lupus disease). Splenomegaly was realized in 15.2% of lupus patients. The abnormal level of liver enzymes was observed in total 48.55% including 8.7%, 5%, and 34.7% abnormal AST, ALT, and both, respectively. The increased level of liver enzymes was categorized into three levels, including liver enzymes less than 100 (23.1%), between 100 and 1000 (23.1%), and more than 1000 (2.1%).

No major and minor organ involvement and lupus signs and symptoms were associated positively with SLE hepatic involvement in this study (*P* > 0.05). The only negative relation was realized between gastrointestinal bleeding and increased level of liver enzymes in lupus patients. Abnormal level of liver enzymes was reported 52.2% and 25.8% in those without and with gastrointestinal bleeding (*P* = 0.007).

Comorbidity was observed in 21.7% of lupus patients (Table 1). The abdominal pain was reported in 37%. Location of abdominal pain in lupus patients was detected mainly in epigastric (25%) following right upper quadrant area. The ascites, pleural effusion, anorexia, weight loss, and nausea/vomiting were present in 16.6%, 12.3%, 10.1%, 17.3%, 13.7%, and 11.6%, respectively. Gastroesophageal endoscopic study was performed in 14 lupus patients with gastrointestinal bleeding (10.14%). Abnormal level of liver enzymes was detected in 5 patients with gastrointestinal bleeding (3.6%). Endoscopic results were positive in all five cases including one patient with peptic ulcer, two cases with gastritis, and two esophagitis cases.

One patient with SLE was presented with pancreatitis in the first admission (0.7%) and one of them has macrophage activation syndrome as first presentation of SLE (0.7%). The prothrombin time test (PT) result and partial thromboplastin time (PTT) were abnormal in 14.5% and 2.9%, respectively. Abnormal PT was corrected with vitamin K injection without any signs and symptoms of liver encephalopathy. Hypoalbuminemia and low total protein were realized in 20.3% and 11.6%, respectively.

Prevalence of positive lupus classification criteria has been reported in Table 2. Skin and renal involvement were the most common clinical involvement in our patients. Anti ds-DNA as a specific immunologic criteria was positive in 63% of patients. The complement (C3, C4, and CH50) test was low in 31.1%.

## 4. Discussion

Liver enzyme abnormalities have been described in various studies in up to 60% of patients with SLE at some point during the course of their disease [7, 14]. Patients with SLE and elevated liver enzymes can interfere with complicated differential diagnosis. In order to ascribe the elevated level of liver enzymes to SLE disease, different factors and liver involving diseases have to be ruled out such as drug hepatotoxicity, autoimmune hepatitis, viral hepatitis, and fatty liver [7, 10, 11].

Assessment of these factors requires the full evaluation of SLE associated diseases which could result in elevated level of liver enzymes including lupus hepatitis and overlap syndrome [10, 11]. Accurate clinical evaluation, serological tests, and often liver biopsy would be crucial in order to prove the abnormal level of liver enzymes as a component of SLE disease. SLE by itself is not usually associated with a significant liver disease. Liver involvement in lupus is often asymptomatic which is referred to as “lupus hepatitis.” It is characterized by a mild increase in serum transaminase levels. In 28–42% of patients, no obvious causes of liver dysfunction were found, and it was thought to be due to lupus itself [10, 11]. Lupus hepatitis usually responds to SLE treatment and has a benign course with no complication and liver enzymes return to normal in lupus-induced hepatitis by steroid therapy [3, 4, 15]. This result was observed in our study as well.

It is still a controversial issue, but a great deal of evidence exists in the literature in favor of this hypothesis that lupus itself is not associated with a severe and progressive liver injury. However, several authors have mentioned SLE as a trigger of subclinical liver involvement [11]. Asymptomatic elevated level of liver enzymes is frequently associated with lupus exacerbation.

Moreover, sometimes there are overlapping profiles with other autoimmune diseases, such as autoimmune hepatitis, acute hemolytic anemia, and primary biliary cirrhosis which could result in higher levels of hepatic enzymes. If there is autoimmune evidence in some organs such as thyroid, hematologic, central nervous system, or joints and raising of liver transaminase, SLE should be considered as the first diagnosis [8, 14]. Isolated autoimmune liver involvement is compatible with autoimmune hepatitis, but these patients should be followed up for complete SLE criteria for several years. These are accompanied by changes in immunological liver markers and autoantibodies that help to establish an accurate diagnosis. Positive anti-smooth-muscle (sm) antibodies and anti-liver-kidney antibody (LKM) are expected in primary autoimmune hepatitis [16]. In a study in pediatrics and adult SLE, the rate of on autoimmune hepatitis was more common in children (9.8% compared with 1.3% in adult patients) [17].

Another cause that must be considered as a contributing factor in lupus hepatitis is drug-induced liver toxicity. It is also common in SLE and may be ascribed to chronic consumption or high doses of medicines used to control autoimmune disorders such as immunosuppressants, anti-inflammatory drugs (steroidal and nonsteroidal), and disease-modifying antirheumatic drugs (DMARDS). Increased rate of drug-induced hepatotoxicity was reported in lupus patients [7, 14]. All medication that was being prescribed in lupus could result in elevated liver enzymes but this complication is uncommon in some drug such as monoclonal antibodies (rituximab, belimumab) and antimalarial antibodies [11]. In a study by Huang et al., 35 cases of drug-induced hepatotoxicity were reported among 1533 SLE patients [18], but in another study, by Takahashi et al., liver involvement by drug-induced injury was reported in 31% of total 123 SLE patients [7]. At the moment, it is impossible to know with certainty whether this high incidence could be the result of chronic lupus drug use, relatively high doses of medicine, and different drugs commonly prescribed to treat this disease, or the presence of any kind of susceptibility that makes these patients prone to drug-induced hepatotoxicity. Drug-induced hepatitis in lupus patients was mainly as lobular, portal, or periportal involvement. In our study, hepatic involvement due to medications was not as high as these studies. It was reported in 2.1% of patients during follow-ups.

SLE patients have elevated levels of systemic oxidative stress that could be obvious in lupus patients with the elevated liver enzyme [19]. The spectrum of liver disease in SLE was assessed in a study by Piga et al. in 2010. Liver enzyme abnormalities were observed in 18.6% of patients. They concluded that liver hepatitis is generally subclinical with a fluctuating course and responds greatly to moderate to high doses of prednisone without progression to end-stage liver disease [20].

In our study, totally 48.55% of childhood lupus cases had hepatic involvement. No significant association was observed between various organs involvement and abnormal level of liver enzymes in children with lupus in our study. But elevated liver enzymes were detected mostly in lupus patients without gastrointestinal bleeding (52.2% without versus 25.8% with gastrointestinal bleeding). In other studies, hepatic dysfunction was reported approximately between 23% and 60% of total SLE patients [3, 4]. According to these studies, hepatomegaly and splenomegaly were reported in one-third and one-tenth of patients, respectively. Jaundice was considered in one-quarter of all cases. Hepatic dysfunction was usually mild and nonspecific similar to results in our study.

Different types of hepatic involvement in various studies included lupus hepatitis, Budd-Chiari syndrome, regenerative nodular hyperplasia, autoimmune hepatitis, drug-induced hepatitis, and primary biliary cirrhosis [8, 11].

Hepatic involvement was mild and asymptomatic in our subjects, except in four lupus cases with overlapping autoimmune hepatitis. They were presenting with increased levels of alkaline phosphatase and gamma-glutamyltranspeptidase as well as specific autoimmune hepatitis antibodies. In these cases, liver enzymes did not reach the normal level after initiation of standard SLE treatment.

In a study in Japan, the hepatic dysfunction was seen at the time of lupus diagnosis in 45% [7]. In this study, neurological involvement was more common among lupus patients with hepatitis rather than those without hepatitis [7]. However, in our study, there was no significant correlation between hepatic and neurological involvement.

The jaundice was not a common finding related to lupus hepatitis and it was considered as an indicator of hemolysis. The rate of jaundice in SLE patients was reported to be 24% in Runyon et al.'s study [14]. In our study, hemolytic anemia (positive coombs test and elevated retic count) was reported in 2.9% of lupus patients, although there is positive coombs test in 14.5% of patients.

Hepatic involvement including hyperbilirubinemia and increased liver transaminases can be the only presentation or one of the main symptoms in neonatal lupus erythematosus (NLE) but the mechanism of NLE is different with SLE [21].

Pancreatitis was observed in one of our SLE patients. Acute pancreatitis is not common in SLE patients especially as a first presentation of SLE but it is associated with sever and fatal coarse of SLE [22, 23]. Prevalence of pancreatitis has been reported 0.9% in juvenile SLE [23]. Another cause of hepatitis in SLE patients is macrophage activation syndrome [24].

Treatment of underlying disease also resulted in subsequent alleviation of abdominal pain and anorexia in all subjects of our study.

Liver biopsy was not performed in all our lupus cases because of later normalization of liver enzymes following initiation of standard treatment of SLE and ethical concern. This was a limitation of this study.

## 5. Conclusion

Approximately half of pediatric subjects with SLE have hepatic involvement. No significant correlation was observed between various organs involvement and abnormal level of liver enzymes. But, it was reported more frequently in lupus patients without gastrointestinal bleeding (about one-third of patients with gastrointestinal bleeding had abnormal level of liver enzymes). Serum transaminases should be routinely checked out in lupus patients in order to rule out other accompanying liver problems, particularly in patients with higher level of liver enzymes.

## Figures and Tables

**Table 1:** Comorbidity in children with systemic lupus erythematosus.

Comorbidity	Frequency
Hypertension	14 (10.1%)
Hypothyroidism	4 (2.9%)
JIA	3 (2.17%)
Diabetes	2 (1.44%)
Myopathy	1 (0.7%)
Sjogren	1 (0.7%)
ITP	1 (0.7%)
Macrophage activation syndrome	1 (0.7%)
Scleroderma	1 (0.7%)

**Table 2:** Frequency of clinical and immunologic criteria in lupus patients.

Clinical criteria	Positive (%)	Immunologic criteria	Positive (%)
Acute cutaneous lupus	57.2%	Positive	93.5%
Chronic cutaneous lupus	5.1%	Positive anti-ds-DNA	63%
Oral or nasal ulcers	23.2%	Positive anti-SM	2.1%
Nonscarring alopecia	2.9%	Positive APL-Ab	8%
Synovitis	39.8%	Low complement	31.2%
Serositis	18.1%	Coombs	14.5%
Renal	51.5%		
Neurologic	33.3%		
Hemolytic anemia	2.9%		
Leukopenia	30.5%		
Thrombocytopenia	21%		

ANA: antinuclear antibody; anti-ds-DNA: double-stranded DNA antibody; anti-SM: anti-Smith antibody; APL-Ab: antiphospholipid antibody.
